# Targeting Mitochondrial Dysfunction in Alzheimer’s Disease Neurons: Lithium Boosts Oxidative Phosphorylation

**DOI:** 10.3390/cells15100896

**Published:** 2026-05-14

**Authors:** Benedict C. Albensi, Aida Adlimoghaddam

**Affiliations:** 1 Department of Pharmaceutical Sciences, Barry & Judy Silverman College of Pharmacy, Nova Southeastern University, Fort Lauderdale, FL 33328, USA; 2 Division of Neurodegenerative Disorders, St. Boniface Hospital Albrechtsen Research Centre, Winnipeg, MB R2H 2A6, Canada; 3 Department of Pharmacology & Therapeutics, Max Rady College of Medicine, University of Manitoba, Winnipeg, MB R3E 0T6, Canada; 4 Department of Neurology, Dale and Deborah Smith Center for Alzheimer’s Research and Treatment, Neuroscience Institute, Southern Illinois University School of Medicine, Springfield, IL 62702, USA; 5 Department of Medical Microbiology, Immunology and Cell Biology, Southern Illinois University School of Medicine, Springfield, IL 62702, USA; 6 Department of Pharmacology, Southern Illinois University School of Medicine, Springfield, IL 62702, USA

**Keywords:** lithium, Alzheimer’s disease, bioenergetics, mitochondrial function, oxidative phosphorylation, neurons

## Abstract

Alzheimer’s disease (AD) is characterized by the accumulation of amyloid beta (Aβ) and neurofibrillary tangles in brain tissue; however, AD is multifactorial, and different etiopathogenic mechanisms involve factors that can affect mitochondrial function, which are associated with AD. While high-dose lithium is a well-established mood stabilizer, accumulating evidence suggests that low-dose lithium provides significant neuroprotection by reversing AD pathology, cognitive impairment, and inflammation. Despite these findings, there is limited information on how lithium affects brain energy metabolism. In the current study, we investigated the effect of lithium (0, 0.1, 1, and 10 mM) on mitochondrial function in AD neurons. Neuronal cells were isolated from the hippocampi of embryonic day 14–17 (E15–E17) control (C57BL/6) mice and 3xTg-AD mice. Mitochondrial oxygen consumption rate (OCR), mitochondrial Cytochrome C Oxidase (COX) activity, total ATP activity, and the expression of mitochondrial complex protein involved in oxidative phosphorylation (OXPHOS) were measured in control vs. 3xTg-AD in the presence and absence of lithium treatment. In the present study, lithium treatment significantly increased (*p* < 0.05) mitochondrial OCR, COX, total ATP, and levels of mitochondrial complex protein subunits (Complex I–V) in 3xTg-AD neurons. However, lithium had no effect on energy metabolism in control neurons. Together, these data indicate that lithium improves mitochondrial function under pathological states. Overall, these results have important implications for the treatment of disorders in which brain energy regulation is compromised, including AD. Particularly, our results highlight a role for lithium in regulating bioenergetics in early-stage AD and suggest that neuronal cells may be a crucial therapeutic target for preventing AD.

## 1. Introduction

Characterized by progressive impairment of cognitive function, Alzheimer’s disease (AD) is a multifactorial neurodegenerative disorder neuropathologically defined by increased neurofibrillary tangles, senile plaques, and extracellular deposits of amyloid-β (Aβ) peptides [1]. Previous research on AD medications and therapeutics has focused on interference with classical hallmarks of AD, such as Aβ plaques [2,3]. However, this methodology has failed to show efficacy in significantly reducing the progression of neurological decline. Accumulating evidence has suggested that mitochondrial dysfunction sets in early in AD progression and is a promising focus for treatment options [4]. Previously, we found that reductions in brain metabolic activity with the lower expression of mitochondrial electron transport components (Complexes I–V) in the 3xTg-AD brain compromise oxidative phosphorylation (OXPHOS) and mitochondrial efficiency by impairing ATP synthesis [5,6,7,8]. Also, other studies reported that cerebral metabolic rate of glucose consumption has been shown to decrease drastically in individuals who have developed AD, possibly due to degradation of the cerebral neuron’s mitochondria [9,10]. Specifically, previous research has found that increasing the neurologic energy consumption rate (metabolism) reduces symptoms of AD in animal models and protects neurons from further degradation [10]. These findings indicate that increasing mitochondrial function in neurons may significantly treat AD.

Drug repurposing is the practice of utilizing pre-existing, FDA-approved drugs for “off-label” purposes [11,12,13]. By utilizing drugs for purposes beyond what they are recommended for, researchers are able to investigate their ability to treat other conditions without many of the risks associated with de novo designs [9,13]. Since these drugs already have safety and toxicology studies with pharmacokinetic profiles, researchers are able to avoid time-consuming early preclinical, phase II, and phase IIa trials due to the adverse drug risks and reactions in humans already being known [9]. While various methods exist for identifying potential drugs, numerous studies have indicated that lithium’s neuroprotective effects may attenuate neurodegenerative effects based on its effects on individuals who have taken it for FDA-approved purposes [14,15]. Clinically, for more than half a century, lithium has been utilized to treat bipolar disorders (BD) [16]. Notably, lithium may act on various pathological processes in AD, as supported by promising findings from both preclinical and clinical studies. Preclinical studies have shown that lithium can reduce Aβ deposition and tau phosphorylation in the PS1xAPP transgenic AD mouse model [10,17,18]. Additionally, previous research has found that lithium-treated (10 mM) neurons had an 87% increase in glucose uptake and increased neural glucose transporter 3, resulting in higher ATP: ADP ratios [10]. A four-fold increase in the rate of glycolysis was also observed when neurons were treated with lithium in vitro [10]. Since neurological deficits associated with AD may be caused by decreased glucose consumption, lithium’s effectiveness in increasing glucose consumption may ultimately be effective in reducing experienced symptoms and pathological advancements of AD conditions. Additionally, lithium modulates inflammation and may reduce the production of pro-inflammatory factors such as IL-1β and TNF-α in AD animal models, thereby attenuating neuronal damage associated with inflammatory responses [14,19]. Other studies indicate that lithium activates mitophagy (mitochondrial autophagy), facilitating the clearance of Aβ and tau pathology and ultimately improving cognitive function in AD [20]. Other neuroprotective mechanisms of lithium include prevention of neuronal apoptosis [21], stimulation of neurogenesis [22], and enhancement of the generation of pluripotent stem cells [23] in various neurodegenerative disorders [24]. Furthermore, structural neuroimaging studies have found that lithium treatment resulted in a volumetric increase in the hippocampus and amygdala in addition to increased thickness of cortical tissue [25]. Several pre-clinical models of AD were able to boost neuron survival, improve memory, and decrease Aβ plaque neuroinflammation (reviewed in De-Paula et al., 2025) [26].

Clinical studies suggest that lithium therapy lowers the risk of AD and helps maintain clinical stability in patients with MCI over a two-year period [27,28]. It has been shown that BD patients receiving long-term lithium therapy exhibit a lower prevalence of dementia, greater cerebral gray matter volume, and improved brain tissue viability compared to patients with other mood-stabilizing drugs [14]. Together, these findings highlight the potential of lithium as a therapy that modifies AD progression. Recent meta-analyses reviewed three randomized placebo-controlled clinical trials involving a total of 232 participants and highlighted the effectiveness of lithium in enhancing cognitive function in subjects with AD and MCI [18]. Aron et al., 2025, recently reported that brain levels of endogenous lithium fluctuate over time and are linked to the maintenance of mental function across the lifespan [15]. Also, their team reported that lithium was significantly reduced in MCI patients [15]. Specifically, these studies compared lithium with recently FDA-approved or under-review therapies, including lecanemab, aducanumab, and donanemab. The findings hypothesize that lithium may offer greater cognitive benefits in AD and demonstrate a more favorable safety profile at lower doses than these drugs [29,30], and its low cost has prompted a reassessment of this conventional medication. These findings have increased medical and pharmacological interest in lithium’s neuroprotective effects and its potential relationship with neurodegenerative diseases. Although the exact mechanism by which lithium acts in AD treatment remains unclear, we suggest that lithium ameliorates AD through improvements in energy metabolism.

Overall, this study aims to assess the validity of the hypothesis that lithium will enhance metabolic function in AD neurons. Mitochondrial respiration rates of neurons isolated from both the control and AD mouse models were assessed using the Seahorse XFe24 Analyzer (Seahorse Biosciences, Billerica, MA, USA) to determine the effectiveness of lithium in sustaining cellular respiration in neurons. Luminescent ATP detection assay kits were then utilized to measure cellular adenosine triphosphate levels in those neurons. Also, cytochrome C oxidase (COX) and citrate synthase activities were assessed spectrophotometrically, and finally, Western blot analysis was conducted to assess levels of OXPHOS protein subunits (Complex I–V) involved in mitochondrial function.

## 2. Materials and Methods

### 2.1. Animals

This study utilizes triple transgenic AD model mice (3xTg) with genotypic expressions of hAPP_Swe_, Hpsi_M146v_, and hTau_301L_ mutations and phenotypic expression of synaptic dysfunction. This strain recapitulates both Aβ and tau pathologies, providing a robust and translationally relevant model to study mitochondrial dysfunction and neuronal bioenergetic alterations in AD. The control samples were derived from wild-type strain (C57BL/6) mice. Samples were taken from male and female embryos at 15–17 days post-conception. All parental mice were provided with *ad libitum* food and water, standard daylight cycle (12 h light, 12 h darkness), room temperature (22 °C), and all other guidelines of the Institutional Animal Care and Use Committee at the University of Manitoba, Winnipeg, MB, Canada, Southern Illinois University School of Medicine, Springfield, IL, USA, and the Institutional Animal Care and Use Committee (IACUC) standards.

### 2.2. Primary Cultures of Neurons

In order to assay the neurons of the subjects, primary hippocampal neurons from both the AD model (3xTg) and the control (C57BL/6) were obtained and prepped according to previous methodologies [31,32]. Briefly, hippocampal neurons from embryonic (E15–7) 3xTg-AD and C57BL/6 mice were cultured. Embryos were obtained following euthanasia under anesthesia (Isoflurane), hippocampal tissues immediately dissected, and submerged in pre-chilled medium containing 139 mM NaCl, 5.36 mM KCL, 0.27 mM Na_2_HPO_4_, 1.1 Mm KH_2_PO_4_, 6.1 mM glucose, and free Hands’ balanced salt solution (HBSS) to obtain an isotonic solution (pH 7.4). Following razor dissection, cells were dissociated by repeated trituration using a Pasteur pipette. The resulting cell suspension was centrifuged at 1000 rpm for 5 min, after which the supernatant was discarded. The cell pellet was gently resuspended by pipetting in warm Neurobasal medium supplemented with B27, and cells were seeded at a density of 3 × 10^5^ cells per well onto poly-D-lysine–coated culture plates. On day 2, cultures were placed on a rotary shaker at 180 rpm for 30 min to remove microglia without excessive stress to the neurons. Further, the media was replaced, and cytosine arabinofuranoside (Ara-C) was added at a final concentration of 2 µM to inhibit proliferation of non-neuronal cells. After 24 h, Ara-C-containing media were removed and replaced with fresh media. Cultures were maintained with partial media changes every 2–3 days. Cells were used for experiments on day 7 in vitro, including metabolic analysis, enzymatic, and molecular assays.

### 2.3. Mitochondrial Respiration Rates Assessment

Cellular respiration rates of neurons isolated from 3xTg (AD) and C57BL/6 (control) mice were measured using the Seahorse XFe24 Analyzer (Seahorse Biosciences, Billerica, MA, USA), as previously described [9,33,34]. The oxygen consumption rate (OCR) was monitored as a series of reagents was added to the plates. The first reagent added was an irreversible ATP synthase (oligomycin, 1 µM), which formed a proton (H^+^) gradient. To assay the maximum respiration of the neurons, an electron transport chain uncoupler (carbonyl cyanide-p-triguromethoxygphenyl-hydrazone also known as FCCP, 1 µM). To arrest cellular respiration and terminate cytochrome c oxidase mitochondrial electron transfer, both rotenone (1 µM) and antimycin (1 µM) were used to inhibit Complex I and Complex III, respectively. Upon completion of the previously described stages, basal respiratory data were calculated and controlled for non-mitochondrial respiration rates as previously described [9,33,34]. Utilizing protein concentrations measured by a colorimetric detergent compatible (DC) protein assay (BioRad, Hercules, CA, USA), the OCR levels were normalized to total protein levels in each well. To assess the maximum respiration rate (MRS), the FCCP-stimulated oxygen consumption rate OCR was divided by the obloigomycin-stimulated OCR. To assess the ability of cells to respond to increased energy production on demand, the spare respiratory capacity is estimated as the difference between the maximal respiratory rate and the controlled basal rate. Coupling efficiency is the ratio of OCR utilized in the creation of adenosine triphosphate (ATP), and it is calculated as approximately the proportion of basal mitochondrial OCR utilized for ATP creation.

Metabolic parameters were measured as follows: Metabolic parameters were derived from oxygen consumption rate (OCR) measurements obtained during sequential Seahorse XF injections. Basal respiration was calculated by subtracting non-mitochondrial respiration (OCR following rotenone/antimycin A treatment) from the basal OCR measured before any drug injection. ATP-linked respiration was determined as the difference between basal OCR and OCR following oligomycin treatment. Proton leak was calculated as the remaining OCR after oligomycin treatment minus non-mitochondrial respiration. Maximal respiration was defined as the FCCP-stimulated OCR after subtraction of non-mitochondrial respiration. Spare respiratory capacity was calculated as the difference between maximal respiration and basal respiration. Coupling efficiency was expressed as the fraction of basal respiration used for ATP production, calculated as ATP-linked respiration divided by basal respiration. All parameters were subsequently normalized to total protein content per well.

### 2.4. Cellular Adenosine Triphosphate Measurement

To accurately assess the amount of ATP produced, a luminescent ATP detection assay kit (ab113849: Abcam, Cambridge, UK) was utilized per manufacturer’s specifications and previously described [6,7,9,33]. Phosphate-buffered saline (PBS) was utilized to wash neurons to remove contaminants. Cells were set to incubate for five minutes on an orbital shaker at room temperature, and then the samples were placed in the dark and allowed to incubate for ten minutes. Further, samples were evaluated on a Microplate Reader (Dynex Technologies, Dockendorf, Germany) at 535/587 nm with control samples that were made with dilutions and underwent the same process described above.

### 2.5. Cytochrome C Oxidase Enzymatic Activity Measurement

Utilizing ScienCell assay kits (828 and 8318, ScienCell Research Laboratory, Carlsbad, CA, USA), cytochrome C oxidase (Utlrospec 2100 pro; GE Healthcare, Chicago, IL, USA) on neurons isolated from 3xTg-AD and control (C57BL/6), with the methodology as stated. To assess COX activity, 550 nm electromagnetic radiation and 412 nm were utilized to assay the synthase activity. All results were normalized densitometry values controlled for protein levels that were assayed with a colorimetric DC protein assay kit (BioRad, Hercules, CA, USA) as described previously [5,6,7,9,33].

### 2.6. Protein Extraction and Western Blot Analysis

Pregnant 3xTg (AD) and C57BL/6 (control) mice were euthanized at E15–17. Hippocampus of embryos was harvested and then homologized in ice-cold radioimmuloprepaitation (RIPA) buffer consisting of the following: 1% triton X-100, 1% phosphate inhibitor cocktail (Sigma-Aldrich, St. Louis, MO, USA), 1% protease inhibitor cocktail (Amresco, Solon, OH, USA), 0.1% sodium dodecyl sulfate (SDS), 150 mM sodium chloride, 50 mM Tris, pH 8.0, and 0.5% sodium deoxycholate. Protein samples were stored at −80 °C prior to Western blotting. Protein concentrations were objectively assayed as previously discussed with a colorimetric DC protein assay kit (BioRad, Hercules, CA, USA). Protein samples were diluted with a 4× Laemmli buffer (40% glycerol, 20% β-mercapothernaol, 16% SDS, 0.01% bromophenol blue, and 0.35 M Tris, pH 6.8) as previously stated (1). Samples were then exposed to 50 °C for 8 min to denature the protein samples. Fifteen micrograms of proteins were loaded into 10% polyacrylamide SDS-PAGE gels (Bio-Rad, Hercules, CA, USA) and underwent electrophoresis at 200 volts for 45 min through Tris-glycine buffer. A ChemiDoc^TM^ MP imager (Bio-Rad, Hercules, CA, USA) activated the gels, and the gels were then transferred to 0.2 µm nitrocellulose membranes (Bio-Rad, Hercules, CA, USA) using the Trans-Blot^®^ Turbo^TM^ Transfer System (Bio-Rad, Hercules, CA, USA). Total proteins on the membranes were assayed with the ChemiDoc^TM^ imager. Upon completing imaging, 0.1% Tween-20 (TBS-T) with 5% milk was utilized to block all membranes except the membranes detecting phosphorylated proteins. Those membranes were blocked with TBS-T with 5% bovine serum albumin for one hour on a tilting platform. As previously mentioned, a series of primary antibodies (Total OXPHOS Rodent WB Antibody Cocktail (ab110413, Abcam, Cambridge, MA, USA, 1:1000 dilution) was added and incubated at 4 °C overnight. Upon completion of the primary antibody, all members were washed three times (fifteen minutes each) with TBS-T buffer. Following this step, the membranes were incubated with 1:2000 dilution of either goat anti-mouse IgG (H + L) antibody. (Jackson ImmunoReesarch Laboratories, West Grove, PA, USA) in TBS-T buffer for one hour at 4 °C with 5% BSA. Finally, the membranes were washed three times for fifteen minutes with TBST-T buffer and prepared by enhanced chemiluminescence ECL (Bio-Red, Hercules, CA, USA) for five minutes. The membranes were visualized utilizing ChemiDoc^TM^ MP imager running Bio-Rad Image Lab software (version 6.1; Bio-Rad Laboratories).

### 2.7. Statistical Analysis

Independent biological replicates, defined as separate neuronal preparations derived from distinct embryos, were used for all analyses (*n*). Data analysis was performed utilizing GraphPad Prism 10 (GraphPad Software) and utilized either two-tailed Student *t*-Test or one-way or two-way ANOVA. For comparative analysis, we used R (v4.5.3; R Core Team, 2026 [35]) with the following packages: bayestestR v. 0.17.0 (Makowski, Ben-Shachar, and Lüdecke 2019 [36]), correlation v. 0.8.8.Statistical significance is established by *p*-values and is demonstrated with * *p* ≤ 0.05, ** *p* ≤ 0.01, *** *p* ≤ 0.001, **** *p* ≤ 0.0001.

## 3. Results

### 3.1. Comparison of Cellular Bioenergetic Profiles in Neurons from C57BL/6 and 3xTg-AD Mice

**Figure 1:**
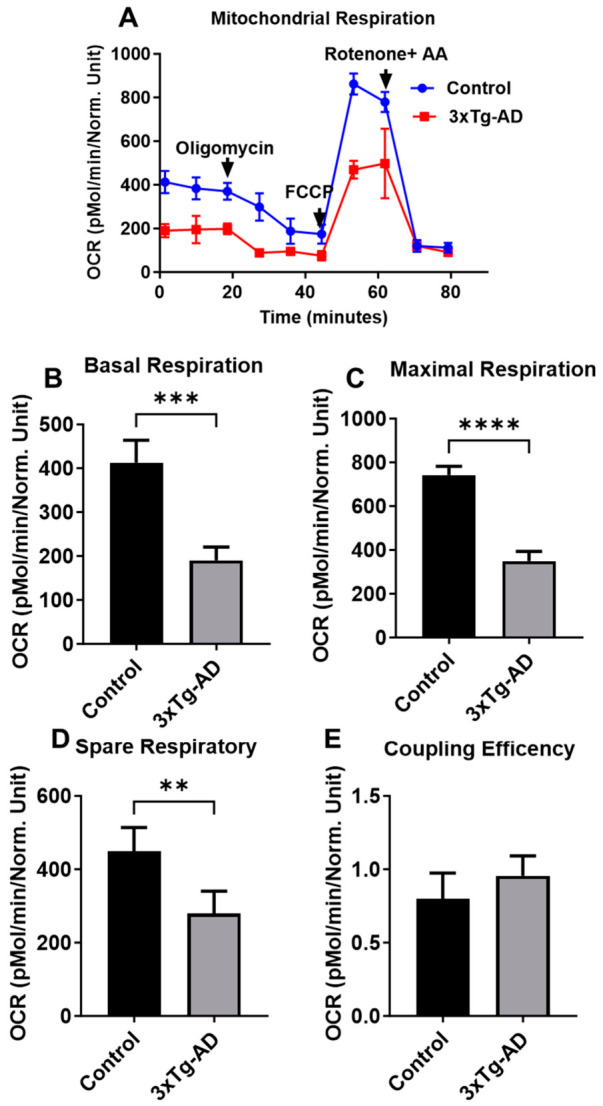
Mitochondrial respiration rates are reduced in 3xTg-AD neurons as compared to C57BL/6. (**A**) Representative kinetic traces showing real-time oxygen consumption rate (OCR) at baseline and following sequential addition of oligomycin, carbonyl cyanide-p-trifluoromethoxyphenyl-hydrazone (FCCP), and rotenone (RR)/antimycin A (AA). OCR was measured in hippocampal 3xTg-AD and C57BL/6-WT neurons using the XF24 extracellular flux analyzer. (**B**) Basal respiration, (**C**) maximal respiration, (**D**) spare respiratory capacity, and (**E**) coupling efficiency were quantified and compared between 3xTg-AD and control neurons. Data are presented as mean ± SD (*n* = 5 per group). Statistical significance was determined using an unpaired two-tailed Student’s *t*-test (** *p* ≤ 0.01, *** *p*  ≤  0.001, or **** *p*  ≤  0.0001).

The mitochondrial oxygen consumption rate (OCR) was measured in neurons derived from hippocampal brain regions from 3xTg-AD and control (C57BL/6) mice (Figure 1A). Basolateral respiration rates in neuronal 3xTg-AD were significantly decreased compared to control groups (Figure 1B). Similarly, OCR associated with maximal respiration capacity was significantly decreased in 3xTg-AD neurons vs. controls (Figure 1C). Consistent with changes in parameters associated with basal and maximal respiration, spare respiratory capacity was significantly lower in 3xTg-AD neurons vs. controls (Figure 1D). However, coupling efficiency was not significantly different in wells containing 3xTg neurons as compared to controls (Figure 1E).

### 3.2. Effects of Lithium on Mitochondrial Bioenergetics and Total ATP Level in C57BL/6 and 3xTg-AD Neurons

**Figure 2:**
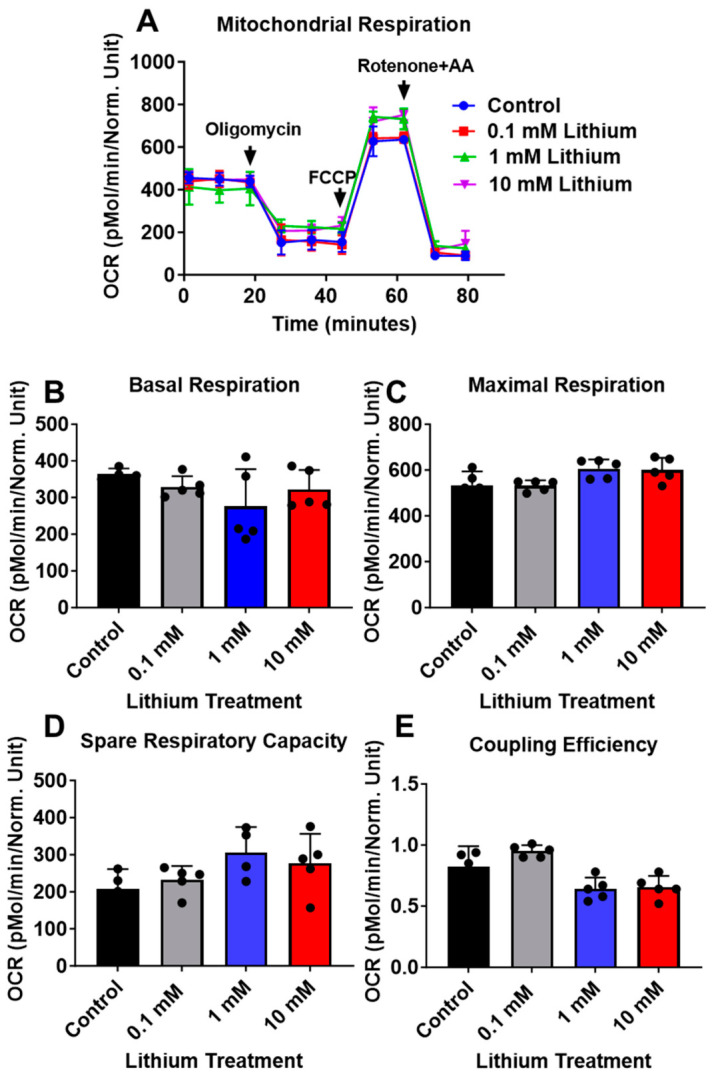
Lithium does not affect mitochondrial respiration in C57BL/6-WT neurons. (**A**) Representative real-time OCR kinetics measured at baseline and after sequential addition of oligomycin, carbonyl cyanide-p-trifluoromethoxyphenylhydrazone (FCCP), and rotenone/antimycin A (AA). Oxygen consumption rate (OCR) was measured in C57BL/6-WT neurons using the XF24 extracellular flux analyzer following 24 h treatment with lithium Chloride (0, 0.1, 1, and 10 mM). (**B**) Basal respiration, (**C**) maximal respiration, (**D**) spare respiratory capacity, and (**E**) coupling efficiency were quantified and compared between vehicle-treated and lithium-treated neurons. Data are presented as mean ± SD (*n* = 5 per group). Statistical analysis was performed using one-way ANOVA, followed by Dunnett’s multiple comparison test.

To evaluate the effects of lithium on mitochondrial bioenergetics, neuronal cells derived from C57BL/6 and 3xTg-AD mice were treated with various concentrations of lithium chloride (0, 0.1, 1, and 10 mM) for 24 h. Lithium treatment did not alter basal respiration, maximal respiration, spare respiratory capacity, or coupling efficiency in C57BL/6 neurons (Figure 2A–D).

**Figure 3:**
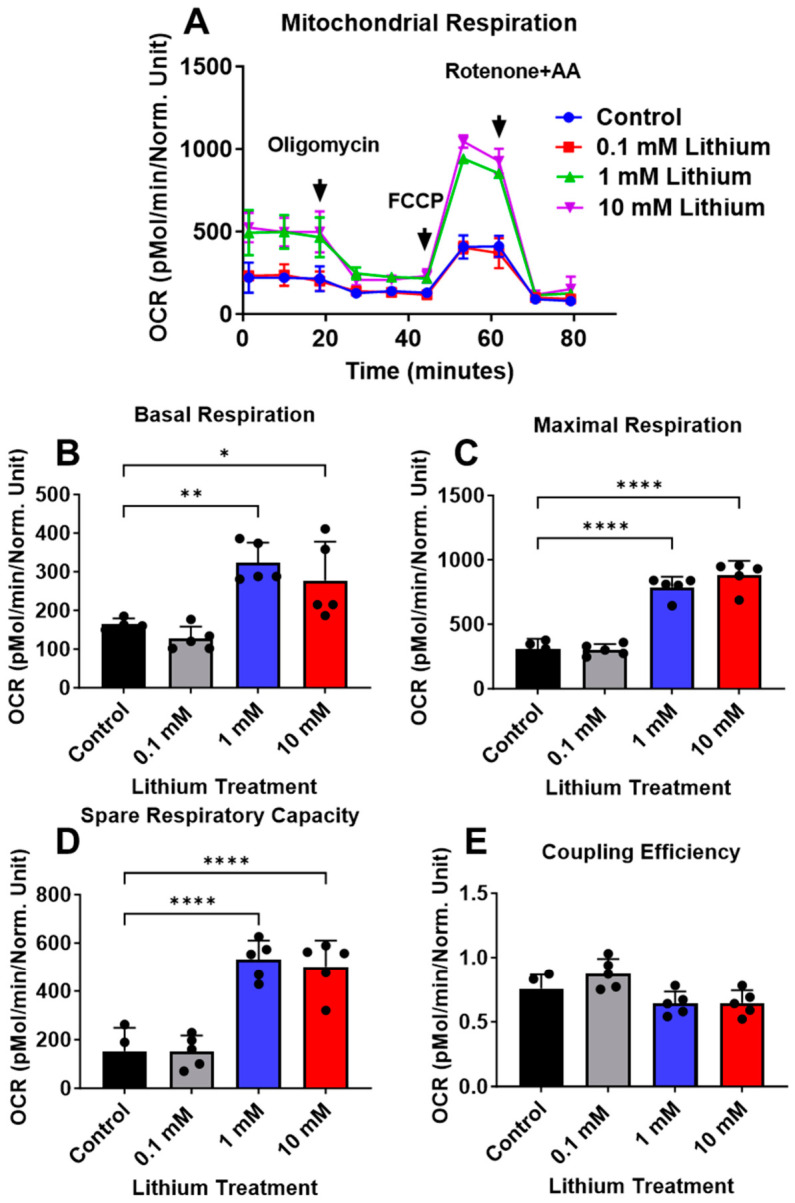
Lithium treatment enhanced mitochondrial respiration rates in 3xTg-AD neurons. (**A**) Representative real-time OCR kinetics measured at baseline and after sequential addition of oligomycin, carbonyl cyanide-p-trifluoromethoxyphenylhydrazone (FCCP), and rotenone/antimycin A (AA). Oxygen consumption rate (OCR) was measured in 3xTg-AD neurons using the XF24 extracellular flux analyzer following 24 h treatment with lithium Chloride (0, 0.1, 1, and 10 mM). (**B**) Basal respiration, (**C**) maximal respiration, (**D**) spare respiratory capacity, and (**E**) coupling efficiency were quantified and compared between vehicle-treated and lithium-treated neurons. Data are presented as mean ± SD (*n* = 5 per group). Statistical analysis was performed using one-way ANOVA, followed by the Dunnett’s multiple comparison test (* *p* ≤ 0.05 or ** *p* ≤ 0.01 or **** *p* ≤ 0.0001).

**Figure 4:**
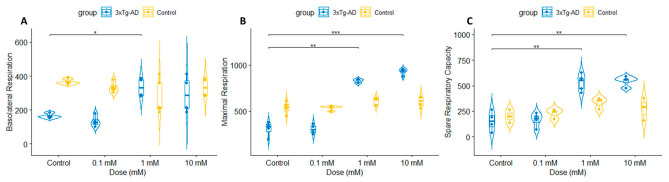
Respiratory function differed across genotypes and showed dose-dependent responses to lithium treatment. Basal respiration (**A**), maximal respiration (**B**), and spare respiratory capacity (**C**) were measured in control and 3xTg-AD neurons treated with different lithium concentrations (0, 0.1, 1, and 10 mM). Statistical analyses were performed using R (v4.5.3; R Core Team, 2026 [35]). (* *p* ≤ 0.05 or ** *p* ≤ 0.01 or *** *p* ≤ 0.001).

**Figure 5:**
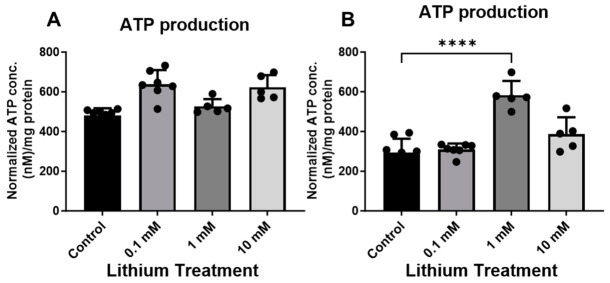
Lithium significantly enhanced ATP levels in 3xTg-AD neurons. Total ATP levels were measured in neurons derived from C57BL/6 mice (**A**) and 3xTg-AD (**B**) mice under control conditions and following lithium treatment (0, 0.1, 1, and 10 mM). Data are presented as mean ± SD (*n* = 5 per group). Statistical significance was determined using one-way ANOVA, followed by Dunnett’s multiple comparison test (**** *p* ≤ 0.0001).

In contrast to C57BL/6 neurons, lithium significantly enhanced mitochondrial bioenergetics in 3xTg-AD neurons. Treatment with lithium for 24 h increased mitochondrial OCR in a dose-dependent manner, with a significant effect observed at 1 and 10 mM (Figure 3A). Basolateral respiration was significantly elevated in 3xTg-AD neurons treated with 1 and 10 mM lithium compared with untreated neurons (Figure 3B). In addition, maximal respiration and spare respiratory capacity were significantly increased following lithium treatment (Figure 3C–D), whereas coupling efficiency remained unchanged (Figure 3E). Respiratory parameters were compared across genotypes and different concentrations of lithium, revealing dose-dependent differences (Figure 4A–C). Consistent with the OCR findings, total ATP levels were not significantly altered in lithium-treated control neurons (Figure 5A), but were increased in 3xTg-AD neurons (Figure 5B).

### 3.3. Effects of Lithium on Mitochondrial-Associated Proteins (OXPHOS) and Cytochrome C Oxidase (COX) in C57BL/6 and 3xTg-AD Neurons

**Figure 6:**
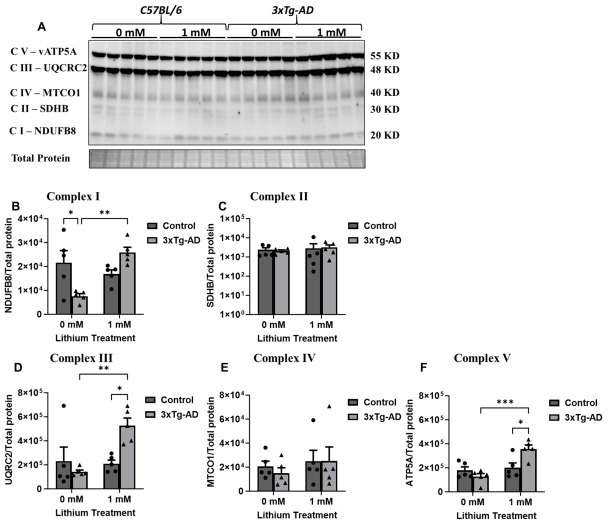
Lithium enhances mitochondrial protein expression in 3xTg-AD neurons. Mitochondrial respiratory complex protein levels were assessed in neurons derived from C57BL/6 and 3xTg-AD mice under basal conditions and after lithium treatment (0 and 1 mM). Data are presented as mean ± SD (*n* = 5 per group). (**A**) Representative immunoblots of Complex I (NDUFB8), Complex II (SDHB), Complex III (UQCRC2), Complex IV (MTCO1), and Complex V (ATP5A). (**B**–**F**) Densitometric quantification of Complex I–V protein levels normalized to total protein. Data are presented as mean ± SD (*n* = 5 per group). Statistical significance was determined using two-way ANOVA followed by Tukey’s multiple comparisons test (* *p* ≤ 0.05, ** *p* ≤ 0.01, or *** *p* ≤  0.001).

**Figure 7:**
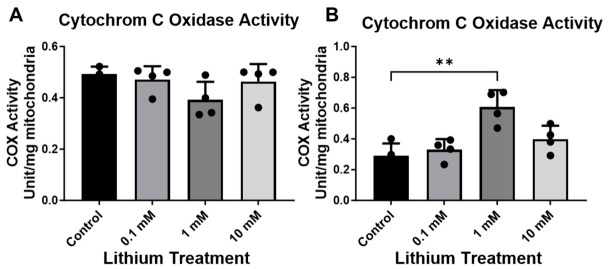
Lithium exposure significantly affects COX activity in AD neurons. Cytochrome c oxidase (COX) activity was assessed in neurons derived from C57BL/6 (**A**) and 3xTg-AD (**B**) mice under basal conditions and after lithium treatment (0, 0.1, 1, and 10 mM). Data are presented as mean ± SD (*n* = 5 per group). Statistical analysis was performed using an unpaired Student’s *t*-test (** *p* ≤ 0.01).

Since the most pronounced effects of lithium were observed in neurons at 1 mM, we focused exclusively on examining the impact of lithium (0 and 1 mM) on the expression of mitochondrial complex subunits. Consistent with the OCR measurements, protein profiling in C57BL/6 neurons revealed that lithium treatment did not significantly affect mitochondrial respiratory composition. Immunoblot analyses revealed stable expression of electron transport chain subunits spanning Complexes I–V, with no discernible differences between treated and control neurons (Figure 6A–F). Consistent with preserved respiratory protein levels, cytochrome C oxidase (COX) activity was unchanged in C57BL/6 neurons following treatment, remaining equivalent to that observed in untreated cells (Figure 7A). By contrast, 3xTg-AD neurons displayed a significant mitochondrial response to lithium exposure (Figure 6A–F). Lithium treatment led to pronounced increases in multiple respiratory chain components, including NADH dehydrogenase β subunit 8 (Complex I), cytochrome c oxidase subunit 1 (Complex IV), and ATP synthase α subunit (Complex V), relative to untreated 3xTg-AD neurons (Figure 6A–B). This coordinated upregulation was accompanied by a significant elevation in COX enzymatic activity, indicating enhanced mitochondrial oxidative capacity (Figure 7B). In contrast, levels of succinate dehydrogenase subunit B (Complex II) and cytochrome b–c1 subunit 2 (Complex III) remained unchanged, suggesting that lithium selectively modulates specific components of the respiratory chain rather than inducing a global increase in mitochondrial protein expression. Consistent with OCR measurements, COX activity was increased in 3xTg neurons following exposure to 1 mM lithium, suggesting a lithium-induced enhancement of mitochondrial function (Figure 7B).

## 4. Discussion

Accumulating evidence indicates that mitochondrial dysfunction plays a key role in the early stages of AD pathogenesis [7,37,38,39,40]. Accordingly, the present study holds therapeutic relevance for mitochondrial dysfunction-associated diseases, including AD. Mitochondrial activity differs markedly among brain cell populations, reflecting their unique metabolic demands. Previous proteomic analyses have reported that both mitochondrial morphology and function vary across specific brain cell types [41]. Consequently, elucidating mitochondrial dysfunction in AD necessitates a cell-type-resolved investigative framework. Each brain cell represents distinct cellular populations with fundamentally different metabolic strategies. Mitochondrial function in neurons significantly declines with age, and because neurons rely predominantly on oxidative metabolism for ATP production, they are especially susceptible to mitochondrial impairment that promotes reactive oxygen species (ROS) accumulation, bioenergetic failure, and cell death [42]. We observed a significant reduction in mitochondrial oxygen consumption rates (OCR) in AD neurons compared with controls. In contrast, lithium treatment markedly improved mitochondrial function, as evidenced by increased cytochrome c oxidase activity and enhanced oxidative phosphorylation. Moreover, neuronal responses to lithium were associated with elevated ATP production, supporting increased metabolic activity and meeting neuronal energy demands. Synaptic mitochondria are exceptionally sensitive to disruptions in the electron transport chain (ETC), where modest reductions in complex I activity (~25%) and more pronounced impairments in complexes III (>70%) lead to substantial declines in oxygen consumption, ATP generation, underscoring the high energetic vulnerability of synaptic function [43]. The current study shows that lithium upregulated the expression of mitochondrial subunits across complexes I and III, potentially promoting the formation of new synapses. Consistent with our findings, other studies have shown that lithium enhances the activity of complexes I and III in the human frontal cortex [44,45]. Additionally, in induced pluripotent stem cell (iPSC)-derived neural precursor cells, lithium treatment significantly increased ATP-linked OCR, particularly in lithium-responsive patient-derived cells [45]. Also, dysfunction of mitochondrial complex V, often due to mtDNA mutations, can reduce ATP production by approximately 30–90%, severely impairing ATP synthesis and leading to lactic acidosis and progressive neurodegenerative changes [43,46]. Evidence from clinical studies in BP models suggests that lithium can counteract such mitochondrial deficits by promoting mitochondrial biogenesis, boosting mtDNA levels, and increasing the overall number of mitochondria [47,48]. Similar to observations in BP, Lithium treatment increased mtDNA levels in a megakaryoblastic cell line [49]. Lithium modulates brain cell function by enhancing mitochondrial activity, lowering oxidative stress, and inhibiting GSK-3β, a protein linked to cognitive decline. In vitro and in vivo studies show that lithium can reduce α-synuclein accumulation and attenuate neurodegeneration in models of Parkinson’s disease. In addition, lithium confers neuroprotection against oxidative stress–induced cell death in α-synuclein overexpressing experimental models, supporting its role as a potential modulator of disease-related neurotoxicity [50,51]. It has been shown that AD is marked by deficits in mitochondrial electron transport, including diminished ATP production through complex V and reduced mitochondrial oxygen consumption. In line with this, we observed a decrease in complex V expression in AD neurons, which was restored by lithium treatment. A review by Motoi et al. has reported that lithium treatment enhanced autophagic activity and improved mitochondrial quality control in neurons [52]. This was evidenced by increased clearance of damaged mitochondria through mitophagy and reduced cellular stress. These effects were consistent with an mTOR-independent mechanism and were associated with improved mitochondrial turnover and function, alongside a reduction in protein aggregate burden in neurodegenerative conditions.

Consistent with clinical studies, our previous FDG-PET analysis demonstrated a decrease in glucose uptake rate in AD cortical tissues [7]. A clinical study shows that lithium increases the use and uptake of glucose in several brain regions [53]. Also, Gherardelli et al. reported that lithium promotes hippocampal glucose metabolism in the APPSwe/PS1ΔE9 (APP/PS1) mouse model of AD [10]. In 3xTg-AD brain tissues, we observed a correlation between glucose uptake rates, ATP, and the capacity of the oxidative phosphorylation (OXPHOS) machinery [7,9,34]. We also found that lithium enhances both ATP production and OXPHOS. Interestingly, previous studies have reported that lithium can either stimulate or inhibit ATP synthesis depending on the tissue type [54,55,56]. This apparent discrepancy may reflect lithium’s ability to interact with the Mg^2+^-ATP complex, which can have different effects depending on the tissue [57].

At the supraphysiological level, 10 mM lithium is expected to exceed the buffering capacity of normal ionic and metabolic homeostasis, leading to widespread perturbation of intracellular processes. Rather than acting selectively through canonical lithium-sensitive signaling pathways (e.g., GSK-3 inhibition or inositol-related mechanisms), 10 mM lithium likely exerts nonspecific effects on membrane potential stability, ion gradients, and mitochondrial integrity. These disturbances can secondarily compromise electron transport chain efficiency, disrupt proton gradient maintenance, and reduce ATP synthase activity, culminating in global energetic failure. Importantly, such concentrations are far above clinically achievable brain levels and therefore do not reflect therapeutic exposure conditions. Instead, they serve experimentally as a “stress test” of mitochondrial resilience, revealing the threshold at which mitochondrial bioenergetics transitions from adaptive modulation at low-to-moderate concentrations (0.1–1 mM) to overt dysfunction under extreme ionic load. The observed suppression of OCR and ATP production, together with reduced cytochrome c oxidase activity, is consistent with a state of mitochondrial stress characterized by impaired electron transport chain flux and reduced respiratory capacity rather than targeted lithium pharmacodynamics. In this context, the 10 mM condition should be interpreted as defining the upper boundary of mitochondrial tolerance to lithium exposure in vitro, rather than representing a biologically or clinically relevant dose.

**Figure 8:**
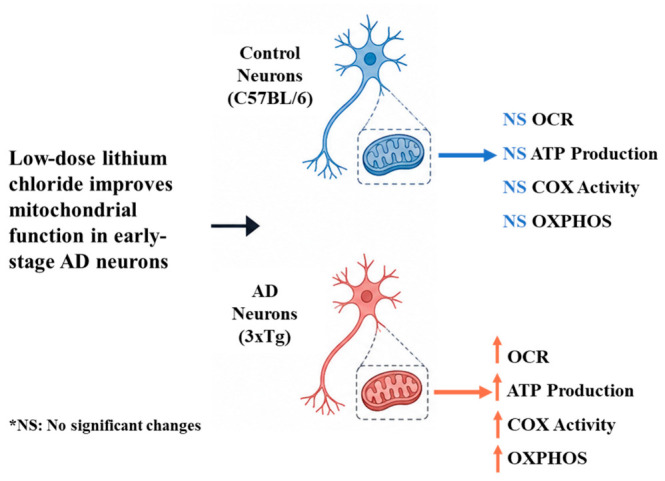
Low-dose lithium chloride improves mitochondrial function in early-stage AD neurons. Low-dose lithium chloride enhances mitochondrial respiratory function, ATP levels, cytochrome c oxidase (COX) activity, and the expression of oxidative phosphorylation (OXPHOS) complex proteins in early-stage Alzheimer’s disease (AD) neurons compared with untreated controls, indicating improved cellular bioenergetics.

Together, these findings (Figure 8) underscore the therapeutic relevance of lithium for disorders characterized by impaired brain energy regulation, including AD. Our results identify lithium as a modulator of mitochondrial bioenergetics in the early stages of AD, with neurons emerging as a critical therapeutic target for disease prevention. By enhancing OXPHOS activity and cellular energy metabolism (OCR and ATP), this work advances the understanding of mitochondrial dysfunction in AD, although further studies are required to elucidate the underlying metabolic mechanisms.

Our analyses were limited to selected metabolic markers; Western blotting for other proteins of interest was not performed, and we did not evaluate synaptic structure or function, which are important readouts of disease progression. Also, in this study, we focused exclusively on neurons to specifically examine lithium’s direct effects on neuronal bioenergetics. Other brain cell types, such as astrocytes and microglia, which are also critical regulators of energy homeostasis and contributors to AD pathology, were not assessed and warrant further investigation. Future studies using adult or aged neuronal models that include neuronal-glial interactions and in vivo, longitudinal approaches are necessary to determine whether lithium treatment can improve cognitive and behavioral outcomes. Overall, our findings provide mechanistic insight into the effects of lithium on neuronal mitochondrial function and bioenergetics and support further investigation in more translational models. We specifically note that additional studies in vivo, including behavioral and clinical investigations, are necessary to determine whether the mitochondrial benefits observed in the present study translate into meaningful therapeutic outcomes in AD and other disorders characterized by impaired brain energy metabolism.

## Data Availability

The original contributions presented in this study are included in the article. Further inquiries can be directed to the corresponding authors.
